# Atomically informed nonlocal semi-discrete variational Peierls-Nabarro model for planar core dislocations

**DOI:** 10.1038/srep43785

**Published:** 2017-03-02

**Authors:** Guisen Liu, Xi Cheng, Jian Wang, Kaiguo Chen, Yao Shen

**Affiliations:** 1 State Key Lab of Metal Matrix Composites, School of Materials Science and Engineering, Shanghai Jiao Tong University, Shanghai, 200240, China; 2 Department of Mechanical Engineering, Stanford University, CA94305, USA; 3 Department of Mechanical and Materials Engineering, University of Nebraska-Lincoln, NE68588, USA; 4 Center for Compression Science, China Academy of Engineering Physics, Mianyang, 621900, China

## Abstract

Prediction of Peierls stress associated with dislocation glide is of fundamental concern in understanding and designing the plasticity and mechanical properties of crystalline materials. Here, we develop a nonlocal semi-discrete variational Peierls-Nabarro (SVPN) model by incorporating the nonlocal atomic interactions into the semi-discrete variational Peierls framework. The nonlocal kernel is simplified by limiting the nonlocal atomic interaction in the nearest neighbor region, and the nonlocal coefficient is directly computed from the dislocation core structure. Our model is capable of accurately predicting the displacement profile, and the Peierls stress, of planar-extended core dislocations in face-centered cubic structures. Our model could be extended to study more complicated planar-extended core dislocations, such as <110> {111} dislocations in Al-based and Ti-based intermetallic compounds.

Prediction of Peierls stress associated with dislocation glide is of fundamental concern in understanding and designing the plasticity and mechanical properties of crystalline materials. Peierls stress (*τ*_*p*_) is the minimum external stress to move a straight dislocation without thermal activation. Atomistic simulations were extensively used to study Peierls stress of dislocations^1^,^2^,^3^,^4^, though having limitations. For example, ab-initio density functional theory (DFT) calculations can accurately calculate dislocation core structure^1^,^2^, but it is computationally expensive or even impossible for studying Peierls stress because of the finite number of atoms in the model. Molecular dynamics/statics (MD/MS) simulations with empirical potentials is capable for predicting dislocation core structure and Peierls stress associated with a glide dislocation^3^,^4^, but reliable empirical interatomic potentials might be unavailable to some complex materials.

Instead, the continuum-scale Peierls-Nabarro (PN) model^5^,^6^,^7^ provides an attractive approach to study Peierls stress of a dislocation, for its simplicity and efficiency in incorporating the nonlinear feature of the dislocation core into the long range elastic fields. In these models, the nonlinear feature of the dislocation core (disregistry profile) is confined in the slip plane, and a constitutive law (i.e. sinusoidal) is developed to describe the atomic interactions across the slip plane. For the region far away from the slip plane, linear elasticity is used to describe stress and strain field caused by the dislocation. In this framework, the dislocation energy comes from two terms, namely the misfit energy in the slip plane and the elastic energy stored in the two half-spaces. The solution to the variational of the dislocation energy is considered as the core structure (disregistry profile). Peierls stress can be obtained by finding the minimum stress to overcome the energy barrier, which was obtained by summing the misfit energy of the atom pairs associated with the rigidly translation of the disregistry profile. In the last several decades, the original PN model^5^,^6^ has been greatly improved^8^,^9^,^10^,^11^,^12^,^13^,^14^. The atomic interactions in the slip plane was better described by incorporating the generalized stacking fault energy or so-called *γ*-surface^10^,^11^, which can be obtained by MD/MS or more accurate ab initio DFT calculations^15^,^16^; the original one dimensional model was extended to two or three dimensional cases^12^,^13^; numerical variational methods were introduced to solve the model instead of the original analytical methods^8^. Of particular importance is the semi-discrete variational Peierls-Nabarro (SVPN) model^9^,^17^, which overcame two major inconsistency of the PN model by discretely summing the atomic misfit energy, and allowing the disregistry profile to relax itself as the dislocation moves under an external stress, in contrast to the original rigid disregistry profile. With these efforts, the SVPN model^9^,^17^ is able to better predict Peierls stress for the dislocation with a compact core, and can also be applied to study dislocation properties under applied stress^18^ and surface effects on Peierls stress^19^.

All these improvements were made under the assumption that the constitutive law and the misfit energy linking the two elastic half-spaces depend only on the disregistry profile at a local site, while ignoring the interactions associated with the large gradient of the nonlinear disregistry in the core region. The original assumption is appropriate within the regions far away from the dislocation core, where the disregistry is almost constant with a negligible gradient. In fact, the nonlocal atomic interactions within the dislocation core have great impact on the dislocation energy^20^. Miller *et al*.^20^ proposed a nonlocal kernel to consider the nonlocal effects in dislocation core and derived its approximate analytical form in real space. They attributed the nonlocal interactions to a modification of the atomic misfit energy, and the nonlocal coefficients were computed by calibrating the misfit energy against the one obtained by atomistic simulations. This method remarkably improves the prediction of the misfit energy, but the core structure did not change much, and sometimes deviated further away from the atomistic results. Schoeck^21^ proposed an average method to calculate misfit energy in around a characteristic distance to account for the large displacement gradient, leading to significant lower Peierls energy and Peierls stress. In this work, we developed a nonlocal SVPN model by incorporating the nonlocal atomic interactions into the SVPN model, wherein the form of the nonlocal interaction energy term is inspired from the nonlocal kernel derived by Miller *et al*.^20^ but extended to multi-dimension, and the nonlocal coefficient is computed directly according to the dislocation core structure. We applied our model to study core structure and Peierls stress of dislocations with planar core in typical face-centered cubic (FCC) metals, such as copper, silver and aluminum, which are of intermediate, low and high stacking fault energy, respectively. The results are validated against MD simulations, and also compared with experiments.

## Results

### Model development

The nonlocal SVPN model is developed by firstly deriving the total energy for a dislocation and then determining the nonlocal coefficients. Total energy associated with a dislocation is a functional of its disregistry vector^8^ **u**(*x*), the relative displacement of the atom pairs across the slip plane perpendicular to the dislocation line sense *x* with respect to the perfect crystal. The disregistry vector generally has three components *u*_*k*_, *k* = 1, 2, 3 denoting the disregistry profiles along the *x-, y-*, and *z-* directions respectively. Incorporating the nonlocal atomic interactions^20^ into the dislocation energy functional of local SVPN model^9^,^17^, the energy functional is written as:


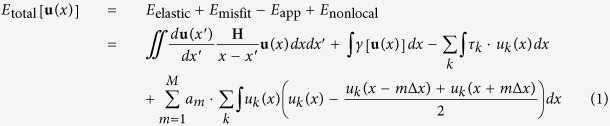


where the four terms on the right hand correspond to the elastic energy stored in the two half-spaces^7^,^8^, the atomic misfit energy in the glide plane^7^, the work done by the external applied stress^9^,^17^, and the nonlocal interaction energy to capture the large slip gradients in dislocation core^20^, respectively. The first three energy terms are the bases of the SVPN model^9^,^17^, where **H** is the anisotropic Stroh tensor^22^,^23^, depending on the dislocation line direction and elastic constants; *γ*[**u**(*x*)] is the generalized stacking fault energy; *τ*_*k*_ is the applied shear stress in the slip plane along the *k* direction. The last nonlocal energy term is an extension of the original one dimensional form ref. 20 by linearly adding the interaction along the *k* direction. *a*_*m*_ is the nonlocal coefficient, and increasing *m* corresponds to incorporating the interactions further and further away from the local site. ∆*x* is the atomic spacing in the slip plane perpendicular to the dislocation line. The nonlocal energy can be thereafter considered as the interaction of the disregistry at a local atomic site *u(x*) with that at the *m-*nearest neighbor site *u(x* ± *m∆x*), until with the disregistry at the *M*-nearest neighbor *u(x* ± *M∆x*).

Equation (1) is a generalized integral form for the dislocation total energy, as a functional of the three dimensional disregistry vector **u = **(*u*_1_, *u*_2,_ *u*_3_). While for a dislocation with Burgers vector being *a*/2<110> in FCC metals, which always tends to dissociate into two Shockley partials on the close-packed plane {111}, the disregistry vector has only two components, *u*_1_ and *u*_3_, in the *xoz* slip plane, with normal direction being along the *y-*axis. By piecewise linearly interpolating the disregistry profiles at atomic sites *x*_*i*_ (*x*_*i*_ = (*i* − *N*/2) ∙ ∆*x*, with *N* ∙ ∆*x* = 200∆*x* being the cut off distance in dislocation energy calculation, ∆*x* is 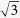
*b*/2 for a screw and *b* for an edge dislocation), the same method used in local SVPN model^9^,^17^ to discretize each energy term, the total energy functional is discretized as:


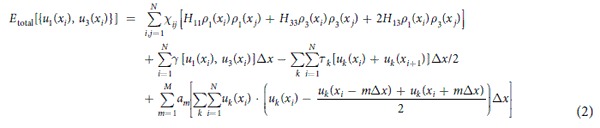


where the discrete coefficient *χ*_*ij*_ for the elastic energy term is,


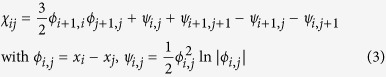


Components of Stroh tensor *H*_*ij*_ for dislocations in typical FCC metals were calculated following anisotropic theory^22^, with elastic constants obtained by molecular statics simulations using embedded-atom-method potential^24^,^25^,^26^ for better comparison with MD simulations. Values for *H*_*ij*_ can be found in ref. 27, which are listed in Table 1 with *H*_13_ = 0. 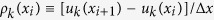 is the dislocation density in the *k* direction. The two dimensional *γ*-surface *γ*[*u*_1_(*x*), *u*_3_(*x*)] is obtained by interpolating between the relaxed γ-surface calculated from molecular statics simulations with quadratic serendipity shape function^28^.

The discretized nonlocal energy term in equation (2) intuitively illustrates the concept of the nonlocal interaction, which is the interaction of the disregistry at a local site *x*_*i*_ with disregistry at the *m*-near neighboring site *x*_*i*_ ± *m∆x*, or more straightforwardly the interaction between the local atom pairs and its *m*-near neighboring atom pairs. Considering the rapid decay of the nonlocal influence, as implied by the fact that *a*_1_ is by far larger than all the other nonlocal coefficients^20^, we simplify the nonlocal energy term by accounting for only the nearest neighbor nonlocal interaction, and setting the secondary nonlocal coefficients *a*_*m*_ (*m* > 1) to be zero. The simplification is also in coincidence with atomistic simulations, where the cut-off distance used in the empirical potentials limits the range of the nonlocal interactions to only the nearest neighbor region. So the simplified nonlocal energy term is:


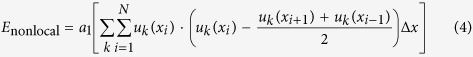


where the only unknown parameter is the nonlocal coefficient *a*_1_ (see Table 1 for all cases studied in this work). They are all directly computed from the dislocation core structure, and the details of the computations can be found in **Methods**. Replacing the nonlocal energy term in equation (2) with the equation (4), the discretized total energy form for a dislocation is derived as:


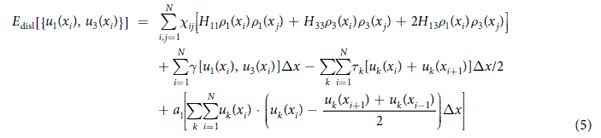


The core structure and Peierls stress of a glide dislocation can be calculated by solving the disregistry vector **u** that minimizes the total energy functional for the dislocation.

### Equilibrium core structure of dislocations under zero applied stress

**Figure 1:**
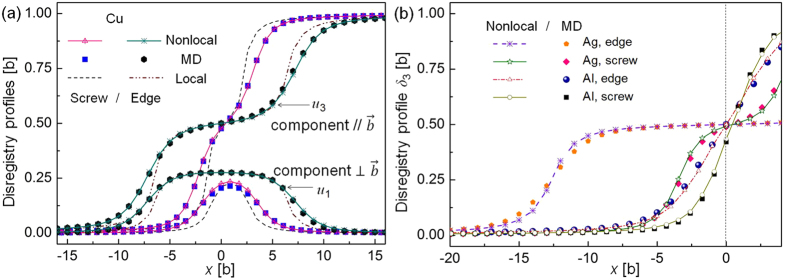
Fully relaxed disregistry profiles at zero applied stress for the 1/2<110> screw and edge dislocations in (**a**) copper and (**b**) silver and aluminum, calculated by different methods.

The nonlocal SVPN model can well predict core structures of dislocations that have planar cores. We firstly apply the nonlocal SVPN model to calculate core structure of dislocations in copper, which has an intermediate stacking fault energy. Figure 1a shows the fully relaxed disregistry profiles for a dislocation with Burgers vector 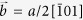 on the (111) plane in copper at zero applied stress. For both the screw and edge dislocations in copper, the two components of the disregistry profiles predicted by the nonlocal SVPN model are all in good agreement with MD simulations, while the local SVPN model^17^ predicted a much narrower core. We further apply our model to dislocations in silver and aluminum, which has relative low and high stacking fault energy, respectively. To better illustrate the difference in their cores, we plot half of the main component (*u*_3_, which is parallel to the direction of the full Burgers vector 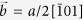) of the disregistry vector predicted by the nonlocal model and MD simulations in Fig. 1b. It clearly demonstrated the improvement of the nonlocal model in predicting the core structure of a dislocation in FCC metals with both low and relative high stacking fault energy.

To further illustrate how the nonlocal energy term affect the core structure, we compare the splitting distance (or the stacking fault width *w*) between the two partials in Table 2 obtained from the local/nonlocal SVPN model, MD simulations and also anisotropic elastic theory^23^ predictions (see **Methods** for details to calculate *w*). Clearly, *w* for all the dislocations predicted by the nonlocal SVPN model are in good agreement with MD results, while it was underestimated by the local SVPN model. Also, the nonlocal model and MD simulations predictions for *w* of the dislocations in copper and aluminum are in better agreement with experiments^29^ or DFT calculations^30^ or MS simulations^31^ with the same potentials used, than the local SVPN model, though the local model is close to the anisotropic theory prediction. While for the dislocations in silver, the nonlocal model and MD predictions for *w* is closer to the theory calculations and the experiments^32^ than the local model. Comparison of *w* in Table 2 straightforwardly demonstrates that the nonlocal energy term improves the core structure for dislocations in FCC with low or high stacking fault energy.

### Prediction of Peierls stress

**Figure 2:**
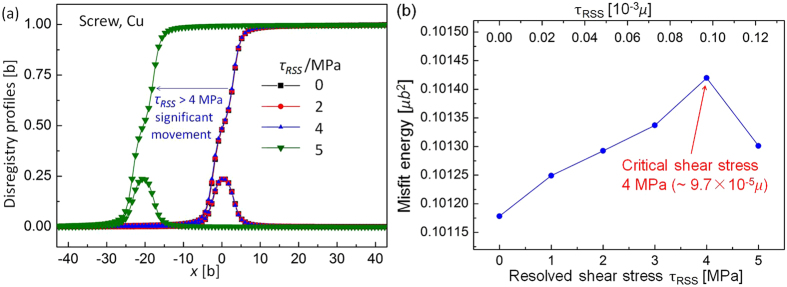
Response of the screw dislocation in copper with increasing resolved shear stress calculated by the nonlocal SVPN model. (**a**) Variations of the disregistry profiles (core structure) under increasing resolved shear stress. Peierls stress, determined as the critical stress to move the screw dislocation, is 4 MPa. (**b**) Variations of the misfit energy as resolved shear stress increases. The critical stress is marked as above which the misfit energy undergoes an abrupt drop.

Peierls stress predicted by both the nonlocal SVPN model and MD simulations is intuitively recognized as a critical resolved shear stress, above which a dislocation is displaced significantly, and below which a dislocation only slightly adjusts its core without obvious forward movement. Figure 2 shows how a screw dislocation in copper responses to increasing resolved shear stress (RSS). Figure 2a explains the critical resolved shear stress is detected as 4 MPa (9.7 × 10^−5^ *μ*), above which the disregistry profiles steps forward about 20*b*. This corresponds to a system instability, which can also be seen from the variations of the misfit energy with increasing RSS as illustrated in Fig. 2b. Before reaching the critical stress, the misfit energy increases as the dislocation is overcoming the Peierls barrier aided by the resolved shear stress, but its misfit energy drops down as long as the dislocation starts to move. This conforms to the impression how the dislocation overcomes the Peierls barrier aided by external stress.

Peierls stress for an edge dislocation in copper is similarly calculated by the nonlocal SVPN model, which is 2 MPa (4.8 × 10^−5^* μ*, see Table 3). Obviously, Peierls stress of the edge dislocation is smaller than that of a screw dislocation, in consistent with its wider core than the mixed dislocation, as can be seen from their core structure in Fig. 1a. More importantly, the Peierls stress of the edge dislocation predicted by the nonlocal SVPN model is much more closer to the experimental value ~0.3 MPa (0.7 × 10^−5^ *μ*)^33^, than the local SVPN model^17^ predicted 69 MPa (1.7 × 10^−3^ *μ*), which was more than two orders of the magnitude.

To better understand the nonlocal effects on Peierls stress, Table 3 lists the Peierls stress predicted by MD simulations and local SVPN model for comparison. Magnitude of Peierls stress given by the nonlocal SVPN model is on the same order of MD results, 1.4 and 1.5 times of the MD calculations for the screw and edge dislocation dislocations in copper, respectively. While the local SVPN model overestimates Peierls stress about 40 to 50 times higher than MD calculations. We further applied our model for predicting Peierls stress of *a*/2<110> screw and dislocations in silver and aluminium, and results are also compared in Table 3. Table 3 shows that Peierls stress predicted by the nonlocal SVPN model is much closer to MD results than the local SVPN model. Compared with the experimental estimations^33^ of Peierls stress for dislocations in FCC, generally around 10^−5^ *μ*, the nonlocal SVPN model is an immense improvement than the local SVPN model. These findings indicate that, the nonlocal SVPN model significantly improves the Peierls stress for the dislocations in FCC metal than the local SVPN model.

## Discussion

**Figure 3:**
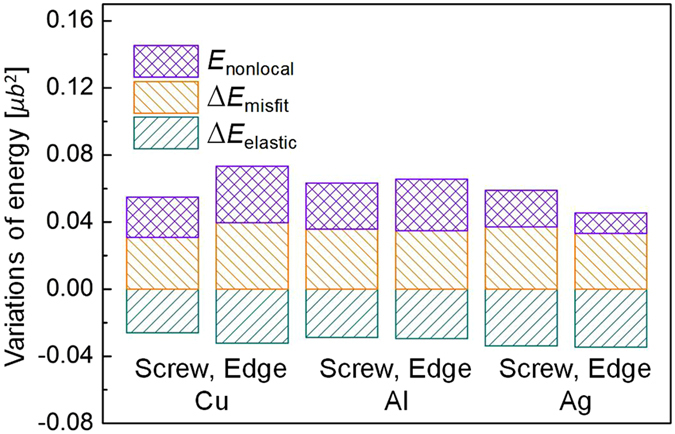
Variations of each energy term for a dislocation predicted by the nonlocal model in this work with respect to those calculated by the local SVPN model^17^ at zero stress.

The nonlocal atomic interaction energy term introduced in the nonlocal SVPN model can smoothen the disregistry profile, and bring the disregistry profiles of dislocations in FCC metals in good agreement with MD simulations, and as a result, improving Peierls stress. To further understand the mechanism, we calculate the variations of each dislocation energy term predicted by the nonlocal SVPN model with respect to the local SVPN model^9^,^17^ in Fig. 3. The misfit energy of all the dislocations predicted by the local SVPN model^9^,^17^ is around 0.1 *μb*^2^. After introducing the nonlocal energy term in the nonlocal SVPN model, the misfit energy increases (positive ∆*E*_misfit_), and elastic energy decreases (negative ∆*E*_elastic_). ∆*E*_misfit_ for all the dislocations studied in this work is around 0.034~0.039 *μb*^2^, which is about 30% increase compared with the local SVPN model. The additional nonlocal energy term *E*_nonlocal_ for dislocations in FCC is about 0.012~0.034 *μb*^2^, which is comparable but smaller than ∆*E*_misfit_.

By combining the original one-dimensional nonlocal Peierls formulation^20^ and the local SVPN model^9^,^17^, the proposed nonlocal SVPN model appears to be a more reliable one. All the results presented above indicate that the nonlocal SVPN model can significantly improve the predictions of both core structure and Peierls stress, and with the predictions being in better agreement with MD calculations and with experimental estimations^33^. The rationale is that, nonlocal SVPN model consolidates the nonlocal atomic interactions^20^ and the advantages of the local SVPN model^9^,^17^, in an effective and simple way. In the framework of SVPN model, most limitations of the original PN model are overcome except the nonlocal interactions, which is compensated for by the additional nonlocal energy term. More importantly, this nonlocal energy term introduced in our model explicitly accounts for the nonlocal interactions in two dimensions, though in a simple linear superposition way, which is is an augment of the original one-dimensional nonlocal Peierls formulation^20^. Moreover, we compute the nonlocal coefficient in a new way, directly from the core structure obtained by atomistic simulations, rather than the original way which calibrates the misfit energy calculated by atomistic simulations^20^. The original treatment of the nonlocal coefficient^20^ leads to the conclusion that, the nonlocal effects can improve the prediction of misfit energy, but cannot change the core structure much, or sometimes predict even more inaccurate core structure compared with atomistic simulations. In contrast, the new approach presented in our model to determine the nonlocal coefficient is physically more realistic, because the nonlocal coefficient should reflect the strength of the nonlocal interactions, and its influences on the core structure.

Last but not least, there is only one nonlocal coefficient that needs to be computed in the nonlocal SVPN model, because we simplified the nonlocal kernel in the original work^20^ to account for the nearest neighbour nonlocal influence, by setting the secondary nonlocal coefficients to be zero. This simplification not only makes it easier to directly compute the nonlocal coefficient from dislocation core structures, but also effectively improves the core structure and Peierls stress for dislocations with planar core, such as dislocations in silver and copper with relative low stacking fault energy, and also dislocations in aluminium with relative high stacking fault energy. This is of special value to apply the nonlocal SVPN model to accurately predict Peierls stress, especially for dislocations with complex core structures. The reasons are as follows: (1) important input of the nonlocal SVPN model including elastic constants and generalized stacking faults energy can be accurately obtained by combing experiments^34^ and *ab initio* calculations^15^; (2) the dislocation dependent nonlocal coefficient can be directly computed from core structures, which can be acquired by experiment techniques, such as high resolution transmission electron microscopy^35^,^36^; (3) the original one dimensional nonlocal formulation is generalized into two dimension in this work, which can also be easily extended to three dimensional condition, and therefore can well describe dislocations with complex core structures.

## Methods

### Nonlocal SVPN model for a dislocation in FCC metal

**Figure 4:**
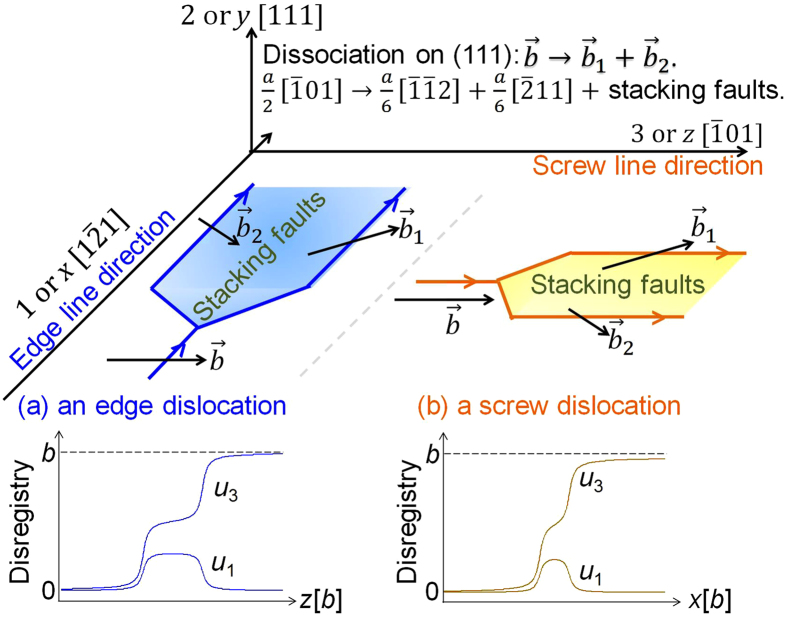
Illustration of a full (**a**) edge and (**b**) screw dislocation dissociating into two Shockley partials connected with stacking faults on the (111) plane in FCC metals.

Coordinate system in Fig. 4 is adopted to present a straight dislocation with Burgers vector 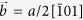 on (111) slip plane in FCC metal, such as copper. It tends to dissociate into two Shockley partials connected with stacking faults. The *x*-, *y*-, and *z* axes are along 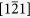, [111] and 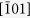 crystalline directions, respectively. For an edge dislocation in Fig. 4, dislocation line is along *x* axis, while the screw dislocation line is along *z* axis, parallel to Burgers vector 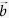. Their core structures can be described by a generally three dimensional disregistry vector **u**(*z*) for an edge dislocation or **u**(*z*) for a screw dislocation, being defined as the relative displacement between the atom pairs across the slip plane, where z (*x*) denotes the coordinate perpendicular to the edge (screw) dislocation line. For simplicity, we use **u**(*x*) to denote **u**(*z*) for the edge dislocation in the energy expressions. Suppose the dislocation is confined in the slip plane, the disregistry vector **u** has two components, *u*_*k*_(*x*), *k* = 1 or 3, representing the disregistry along *x* and *z* axis, respectively. Illustration of the two components of the disregistry vector is shown below the dissociation.

The nonlocal coefficient *a*_1_ is determined in two steps: (1) Calculate the disregistry profiles at any *a*_1_ and zero stress by minimizing the total energy in equation (5), with a Volterra dislocation (step function) taken as the initial trial profile and its values at the two ends are fixed during the energy minimization, same to the approach in local SVPN model^17^. (ii) Find the optimal *a*_1_ that minimizes the sum of the squared errors of the dominating disregistry profile (main component *u*_3_) calculated by the nonlocal model and MD simulations:


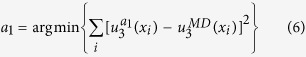


The nonlocal coefficient *a*_1_ determined from equation (6) for the screw and edge dislocations is listed in Table 1.

Core structure and Peierls stress are obtained from the energy functional in three steps. Firstly, compute the equilibrium dislocation core structure under zero stress by minimizing the total energy in equation (5). Secondly, gradually increase the applied stress in equation (5), and calculate the equilibrium disregistry profiles under each glide stress by minimizing the total energy. For the edge and screw dislocations in Fig. 4, only *τ*_*yz*_ (= *τ*_3_ = *τ*_*RSS*_) is applied. We apply the external stress in this way just to simplify the Schmid factor to be one. Finally, find the critical resolved shear stress when the system instability happens, and this critical glide stress is determined as Peierls stress. Note the model input such as components of the Stroh tensor *H*_*ij*_, generalized stacking fault energy surfaces, are all the same with those in ref. 27.

### Molecular dynamics simulations

**Figure 5:**
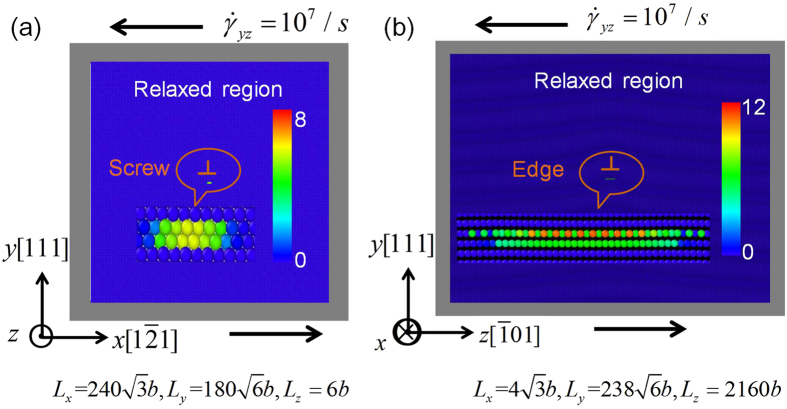
MD simulations model for (**a**) a screw and (**b**) an edge dislocation in copper, where the relaxed region is surrounded by the rigid region in grey color.

The core structures calculated by MD is the calibration criterion used in this work to determine the nonlocal coefficient of the nonlocal SVPN model, and the Peierls stress calculated by MD is to further examine the nonlocal SVPN model. Figure 5a shows a screw dislocation model for MD simulations, including two parts: the relaxed region inside the simulations box, where the atoms are free to move during the relaxation when shear stress is applied, and the rigid region (grey region), which acts as a fixed boundary during the relaxation. Periodic boundary conditions are used along the direction of dislocation line, and the simulation box has three dimensions, *L*_*x*_ × *L*_*y*_ × *L*_*z*_, with the same coordinates in Fig. 4. The dislocation is introduced in the dislocation center according to isotropic linear displacement field^23^, followed by energy minimization to get the fully relaxed dislocation. The relaxed core structure for the screw dislocation at zero applied stress is shown in Fig. 5a, where the atoms are colored by the centro-symmetry deviation parameter^7^. Similarly, an edge dislocation model is shown in Fig. 5b. Apparently, the edge dislocation is much more dissociated than the screw dislocation, as shown as disregistry profiles in Fig. 1.

**Figure 6:**
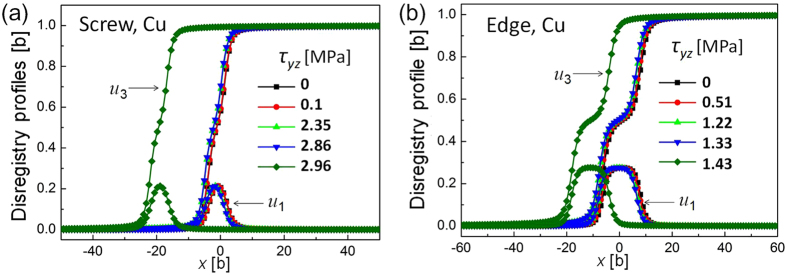
Variation of the disregistry profiles of (**a**) a screw and (**b**) an edge dislocation as the glide stress increases, and Peierls tress determined as the critical glide stress before the dislocation move evidently, is 2.9 MPa and 1.4 MPa, for the screw and edge dislocation in copper, respectively.

To determine Peierls stress, shear stress is applied to the dislocation at a constant strain rate 10^7^/s, followed by minimizing the potential energy after each loading. The relaxed disregistry profiles for the screw and edge dislocations during the shear deformation are plotted in Fig. 6. Peierls stress is determined as the critical resolved shear stress before which the dislocation starts to move evidently in the crystal, being 2.9 MPa and 1.4 MPa for the screw and edge dislocations in copper, respectively.

The dislocation model shown in Fig. 5 can be easily transplanted into screw and edge dislocations in silver and aluminum, with only difference in the lattice constant and the empirical interatomic potentials. Core structure and Peierls stress can be similarly calculated.

### Stacking fault width (*w*) between two Shockley partials

For the local/nonlocal SVPN model and MD calculations, we determine the stacking fault width (*w*) directly from the disregistry profile *u*_3_. For all the dislocations concerned in this work *w* is defined as the distance between the centres of the two Shockley partials, indicated as where the disregistry profile *u*_3_ equals to 0.75 *b* and 0.25 *b*, respectively. We use this definition is because the dissociation of dislocations in aluminium is not obvious due to its relative high stacking fault energy.

Following anisotropic elastic theory^23^, the equilibrium stacking fault width (*w*) between the two Shockley partials can also be calculated as,


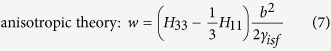


*γ*_*isf*_ is the intrinsic stacking fault energy (see Table 1), *H*_*ij*_ is the component of the anisotropic Stroh tensor (refer to Fig. 4 for the coordinates details and Table 1 for the specific values).

## Additional Information

**How to cite this article**: Liu, G. *et al*. Atomically informed nonlocal semi-discrete variational Peierls-Nabarro model for planar core dislocations. *Sci. Rep.* **7**, 43785; doi: 10.1038/srep43785 (2017).

**Publisher's note:** Springer Nature remains neutral with regard to jurisdictional claims in published maps and institutional affiliations.

## Figures and Tables

**Table 1:** Parameters used in our model.

	*b* (Å)	*μ* (GPa)	*γ*_*isf*_ (mJ/m^2^)	*H*_11_ (GPa)		*H*_33_ (GPa)		*a*_1_ (*μ*/*b*)		
				screw	edge	screw	edge	screw	edge	
Cu	2.556	41.2	44.3	6.05	3.55	3.38	5.94	0.575	0.905	
Ag	2.851	25.6	17.7	3.92	2.21	2.11	3.85	0.523	0.246	
Al	2.892	29.7	129.4	3.68	2.42	2.41	3.68	0.556	0.657	

The values of components of Stroh tensor *H*_*ij*_ in GPa, nonlocal coefficient *a*_1_ in units of *μ*/*b*, for the 1/2<110> dislocations in FCC metals.

**Table 2:** Comparison of the stacking fault width (*w* in units of *b*) for dislocations in FCC metals.

	Cu (screw)	Cu (edge)	Al (screw)	Al (edge)	Ag (screw)	Ag (edge)
Local SVPN	3.72	13.07	1.77	3.97	3.81	17.41
Nonlocal SVPN	5.54	14.75	2.79	5.50	7.43	25.33
MD	5.50	14.40	3.07	5.37	7.54	25.22
Anisotropic	3.90	13.60	1.31	3.17	6.59	25.36
Reference	7.0^29^	14.9^29^	2.6^30^	5.2^31^	~	~29^32^

**Table 3:** Peierls stress for the dislocations FCC metals predicted by the nonlocal SVPN model, MD simulations and previous SVPN models.

		Peierls stress *τ*_*p*_ (in MPa)			
		Nonlocal SVPN	MD	Exp.^33^	Local SVPN (*a*_1_ = 0)
Cu	screw	4	2.9	~0.3	117
	edge	2	1.4		69
Ag	screw	4	3.1	~0.9	125
	edge	1	0.8		54
Al	screw	46	37	~1.4	275
	edge	10	2.6		119
